# Supplementary material for: Heat health risk assessment in Philippine cities using remotely sensed data and social-ecological indicators
Source: Nat Commun. 2020 Mar 27;11:1581. doi: 10.1038/s41467-020-15218-8 (PMC7101384; doi:10.1038/s41467-020-15218-8)
Supplement: Supplementary file 1 — Supplementary Information [file 41467_2020_15218_MOESM1_ESM.docx]

# **Heat Health Risk Assessment in Philippine Cities Using Remotely Sensed Data and Social-Ecological Indicators**

Estoque *et al.*


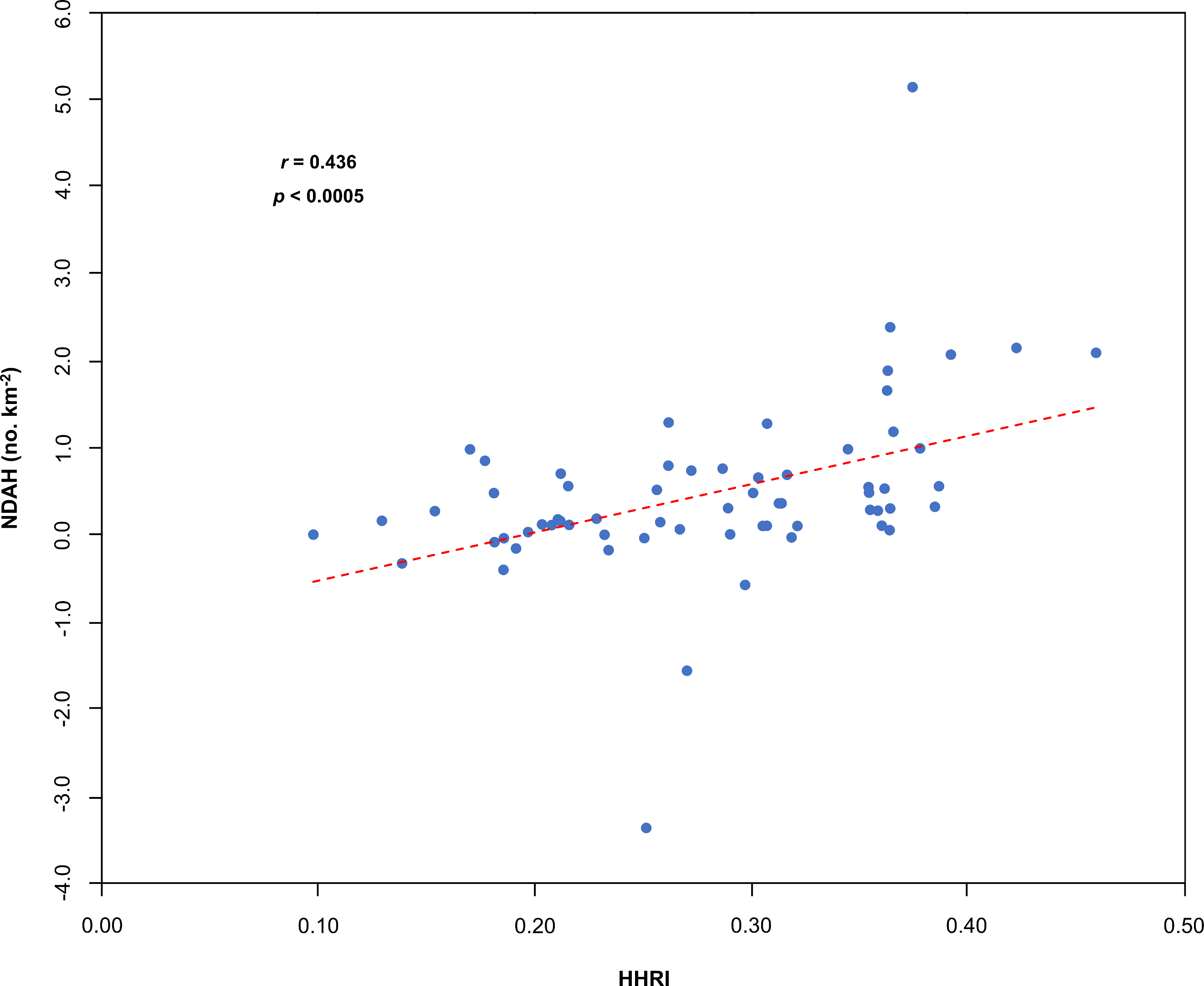


**Supplementary Figure 1** Validation results. The derived heat health risk index (HHRI) had a positive and statistically significant correlation with number of deaths attributable to heat (NDAH) expressed as a density at city level. Each observation (dot) refers to a city (*n* = 65). The area of each city that was used to express NDAH as a density at city level was sourced from the Philippine Statistics Authority^1^. See the “Methods – Validation and sensitivity analysis” section for details.


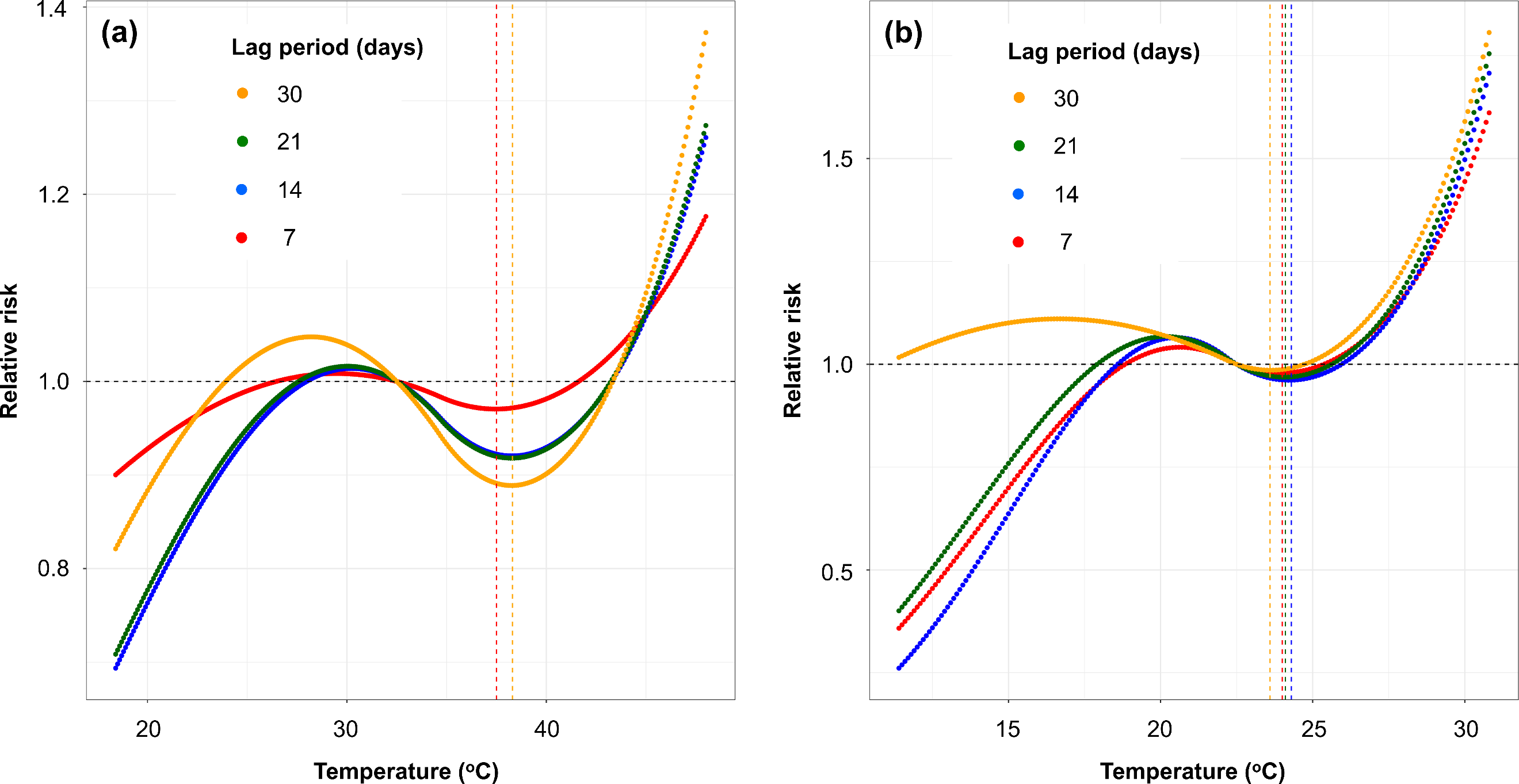


**Supplementary Figure 2** Sensitivity analysis of the number of lag days used to derive the relative risk (RR) curves and minimum mortality temperature (MMT) thresholds. In the daytime (**a**), the MMT (38.3 °C) did not change further beyond lag 14. At nighttime (**b**), the MMT differed slightly among different numbers of lag days, but for consistency, we also used lag 14 to derive the nighttime MMT (24.3 °C). The vertical dotted lines mark the respective MMT values of the different lag periods. Data used: MODIS LST, province-level (32 provinces) daily mortality, and daily average relative humidity (March–May 2006–2011). Data source: MODIS LST data (https://modis.gsfc.nasa.gov), mortality data (Philippine Statistics Authority), and relative humidity data (National Oceanic and Atmospheric Administration’s National Climatic Data Center)

**
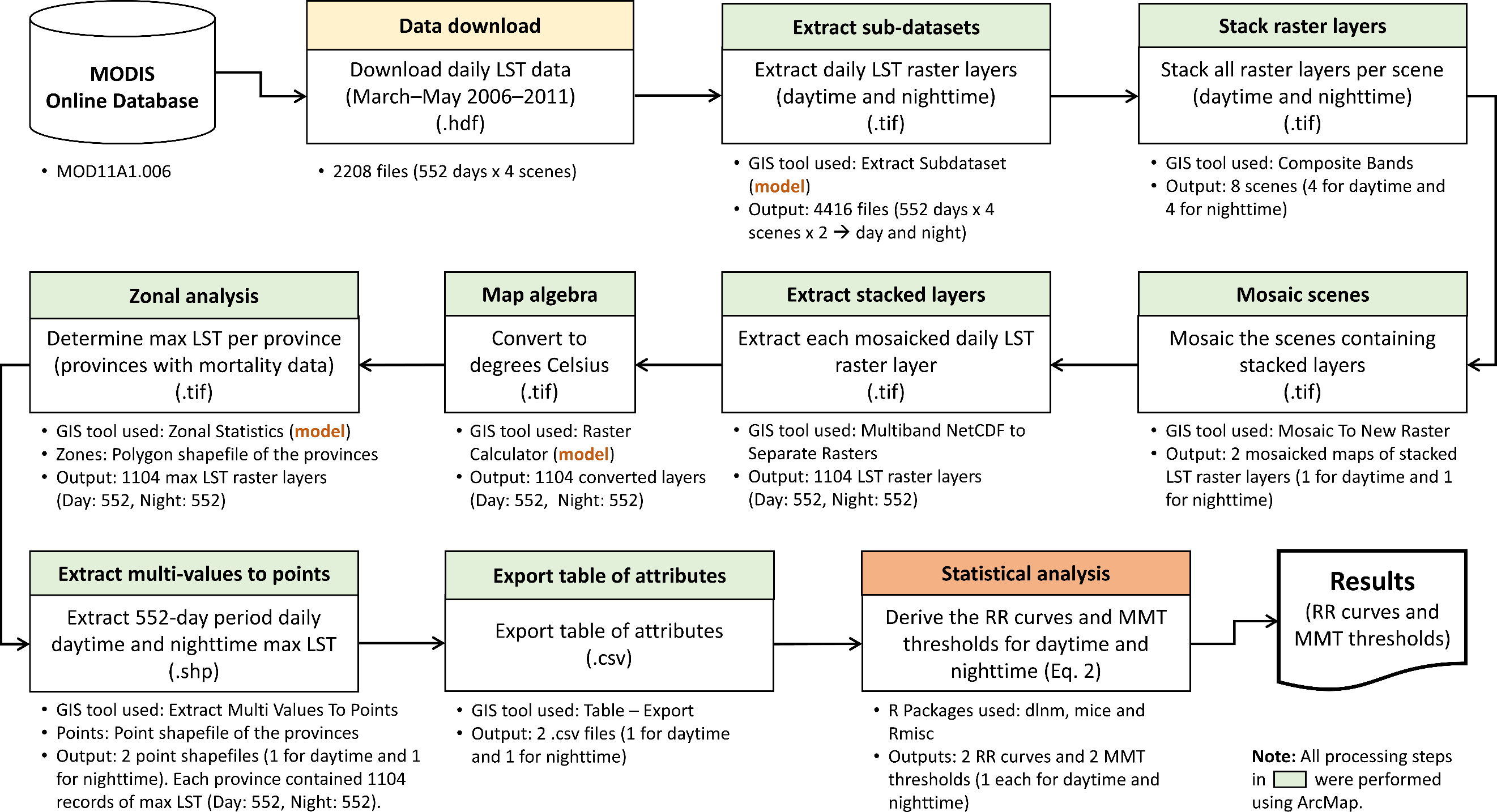
**

**Supplementary Figure 3** Flowchart of MODIS LST (land surface temperature) data processing for derivation of the relative risk (**RR)** curves and minimum mortality temperature (**MMT)** thresholds. In the steps marked with “model”, an iterative raster model was developed for batch processing. Data used: MODIS LST, province-level (32 provinces) daily mortality, and daily average relative humidity (March–May 2006–2011). Data source: MODIS LST data (https://modis.gsfc.nasa.gov), mortality data (Philippine Statistics Authority), and relative humidity data (National Oceanic and Atmospheric Administration’s National Climatic Data Center)


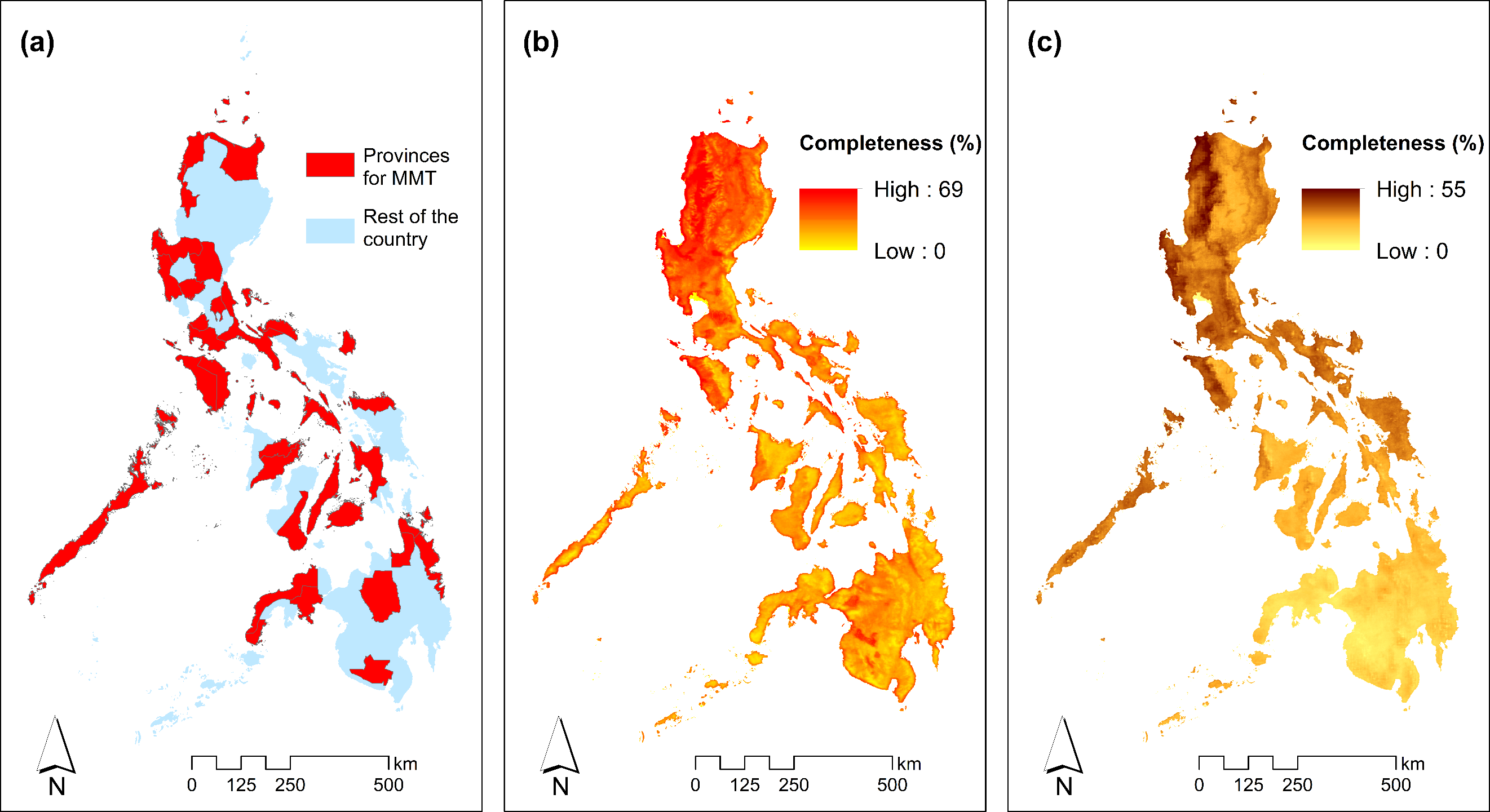


**Supplementary Figure 4** Completeness of the data used to derive the relative risk (**RR)** curves and minimum mortality temperature (**MMT)** thresholds. **(a)** Spatial distribution of the 32 provinces (out of 81) with daily mortality data (March–May 2006–2011) (see Methods for details). **(b)** and **(c)** Pixel-level completeness of the MODIS LST (land surface temperature) data for daytime and nighttime, respectively. Completeness (%) is defined as the ratio percentage of the number of days that a pixel had LST data to the total number of days from March to May of the 2006–2011 period.


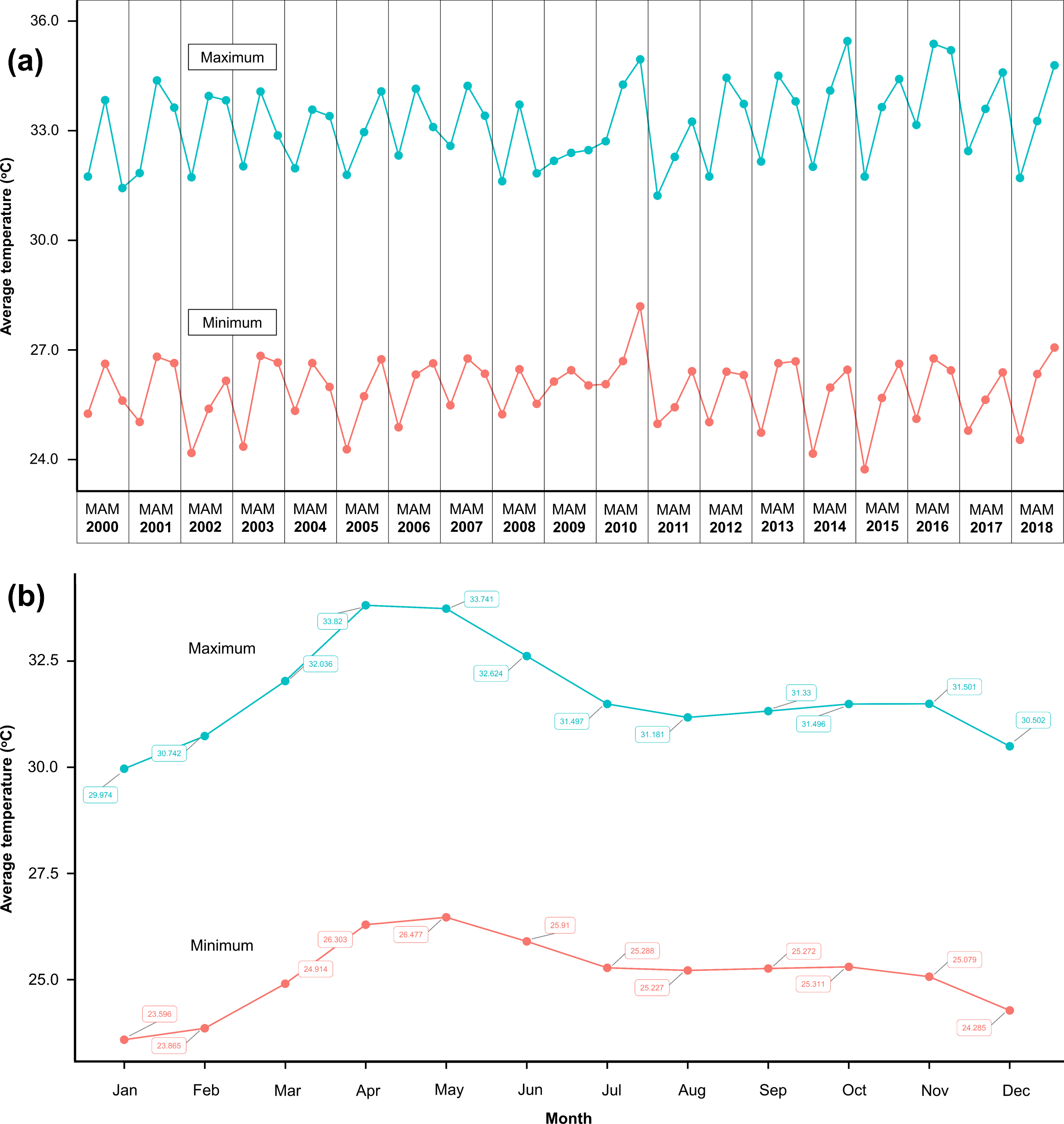


**Supplementary Figure 5** Average maximum and minimum temperature in Manila City, Philippines. **(a)** annual and **(b)** monthly. MAM: March, April, and May (hot dry season). Data used: daily air temperature (2000–2018). Data source: National Oceanic and Atmospheric Administration’s National Climatic Data Center


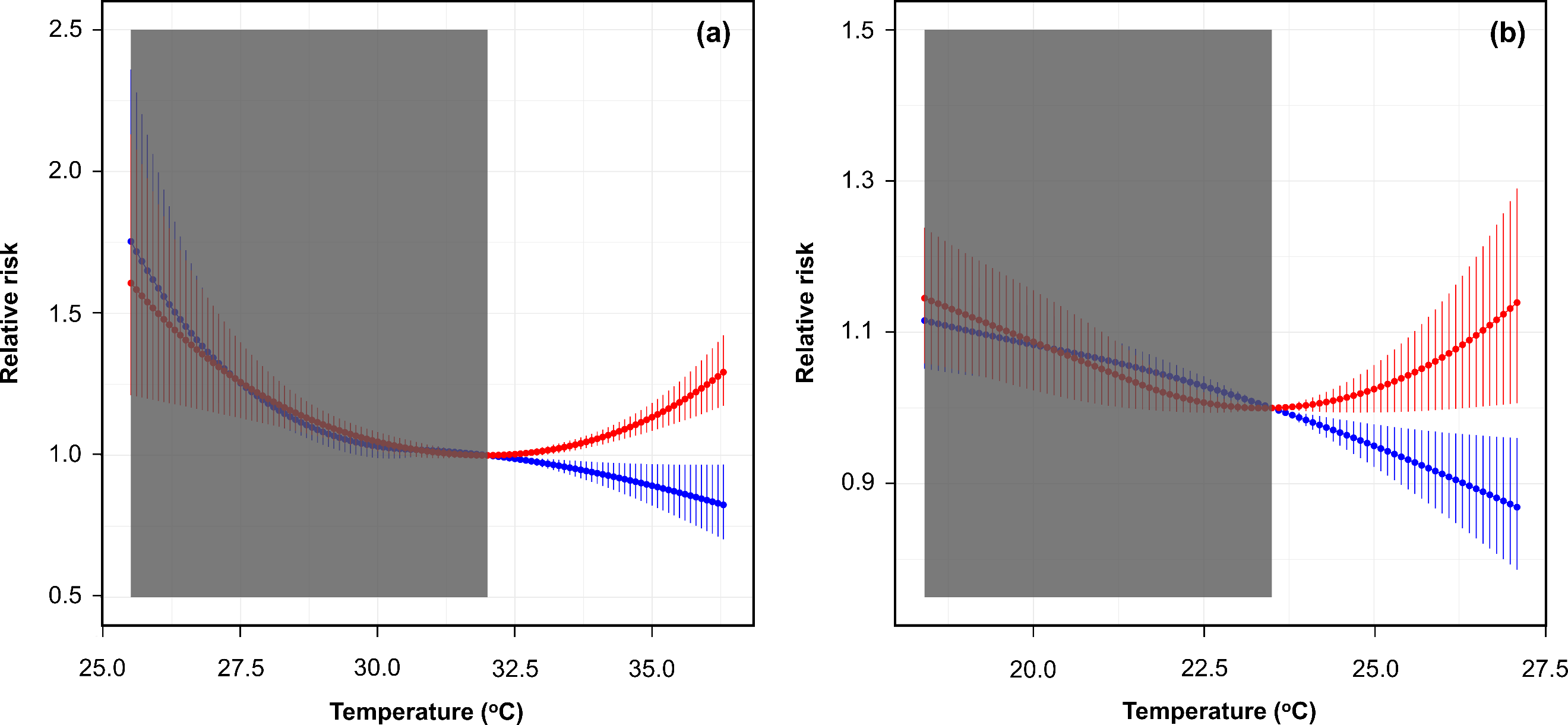


**Supplementary Figure 6** Province-level inter-seasonal comparison of relative risk (RR). **(a)** RR curves based on maximum daily air temperature during the hot dry season (March–May) (red) and hot rainy season (June–August) (blue). **(b)** RR curves based on minimum daily air temperature during the hot dry season (March–May) (red) and hot rainy season (June–August) (blue). Centered on the respective minimum mortality temperature (MMT) thresholds for maximum (32.0 ℃) and minimum (23.5 ℃) daily temperatures, the curves show that the hot dry season (red) had a consistently higher heat-related relative risk as the temperature increased above the MMT thresholds. Data used: province-level (32 provinces) daily mortality, daily air temperature, and daily average relative humidity (March–May, June–August 2006–2011). Data source: mortality data (Philippine Statistics Authority) and relative humidity and air temperature data (National Oceanic and Atmospheric Administration’s National Climatic Data Center)


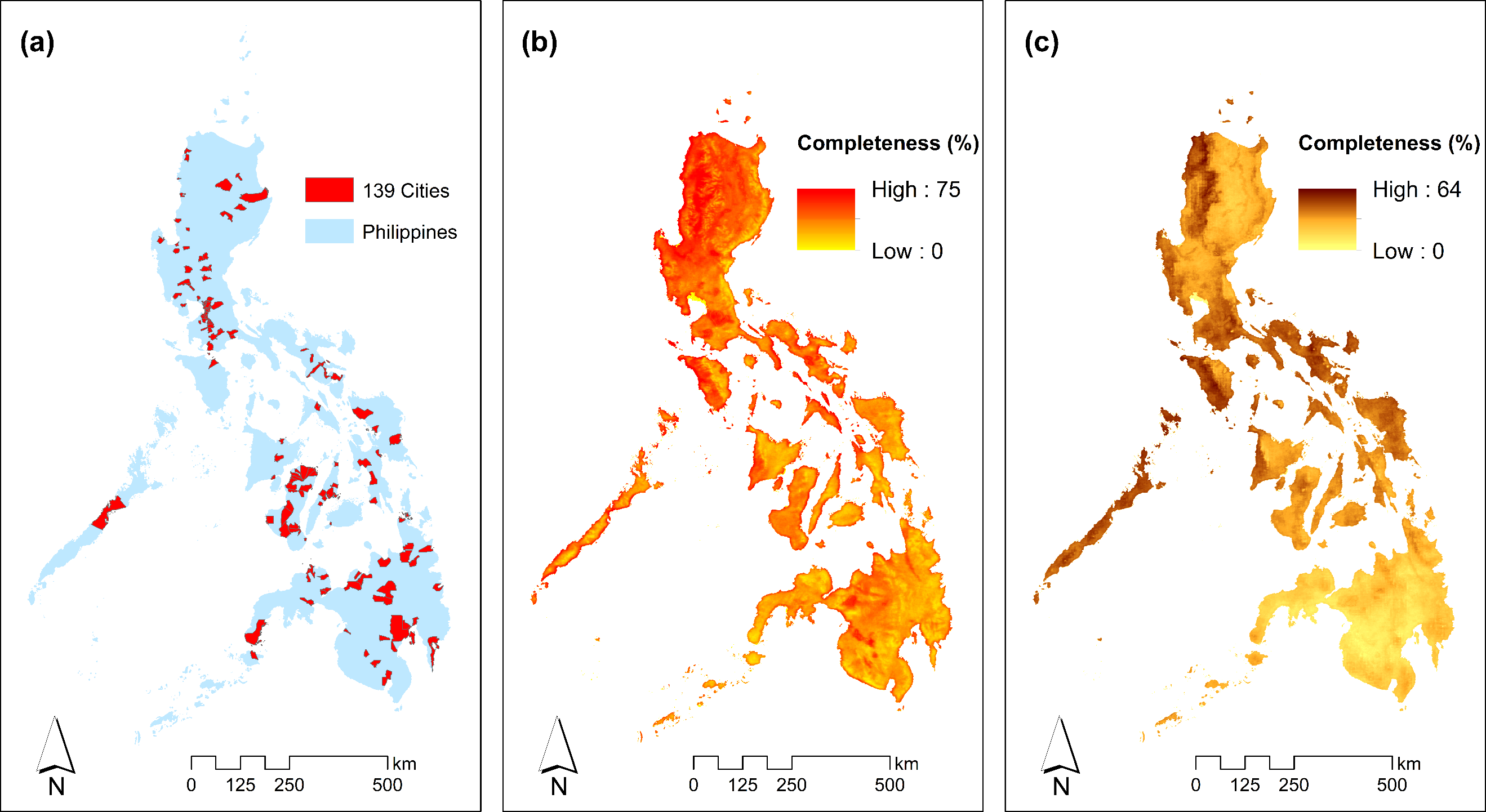


**Supplementary Figure 7** Completeness of the data used to derive the **heat hazard index**. **(a)** Spatial distribution of the 139 cities (out of 145) considered in the analysis (see Methods and Supplementary Table 4 for details). **(b)** and **(c)** Pixel-level completeness of the MODIS LST (land surface temperature) data for daytime and nighttime, respectively. Completeness (%) is defined as the ratio percentage of the number of days that a pixel had LST data to the total number of days from March to May of the 2014–2016 period.


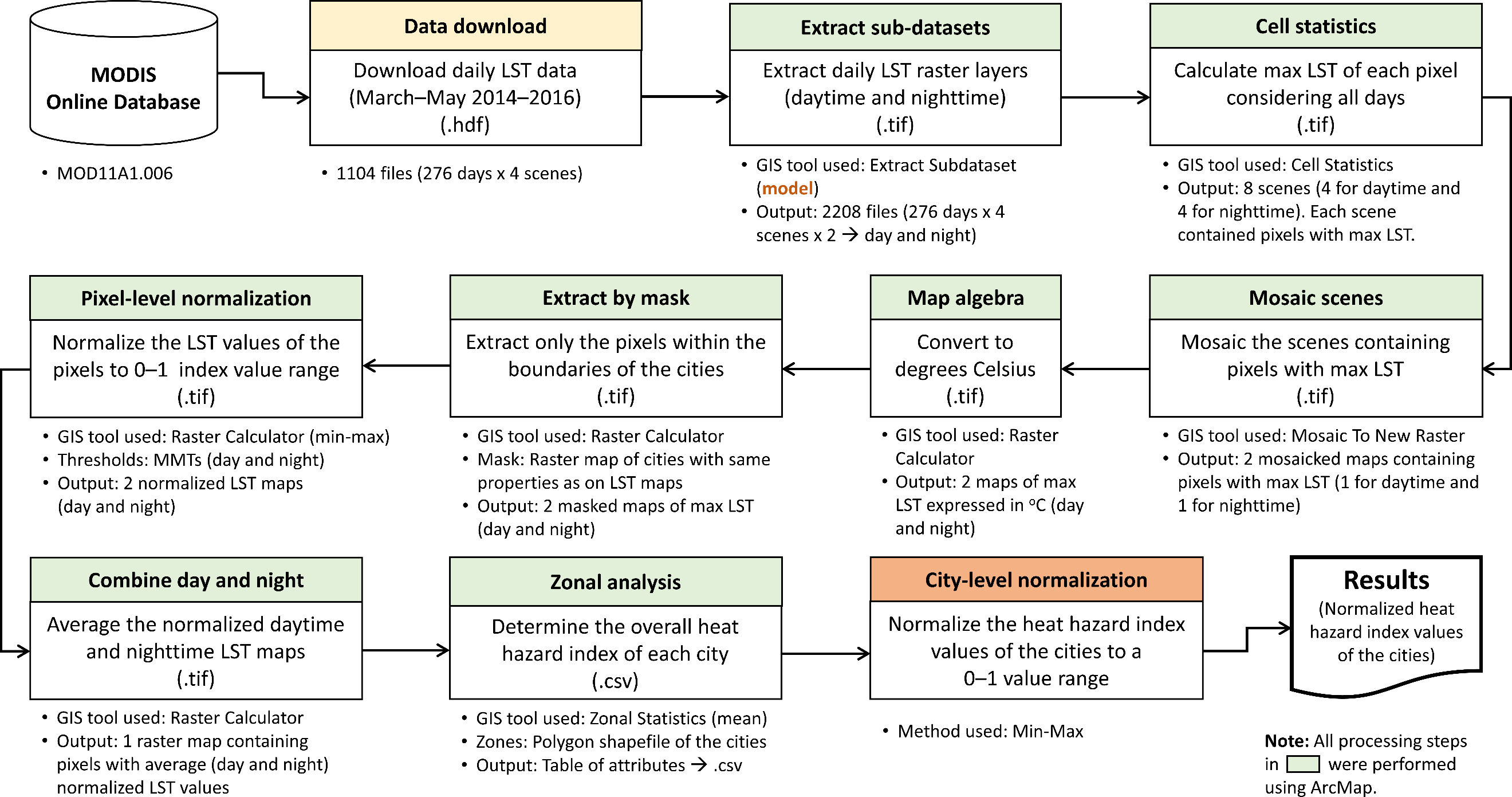


**Supplementary Figure 8** Flowchart of MODIS LST (land surface temperature) data processing for derivation of the **heat hazard index**. In the steps marked with “model”, an iterative raster model was developed for batch processing. MMT: minimum mortality temperature. Data used: MODIS LST (March–May 2014–2016) and MMT thresholds. Data source: MODIS LST data (<https://modis.gsfc.nasa.gov>) and MMT thresholds (this study’s own results – see Fig. 1)


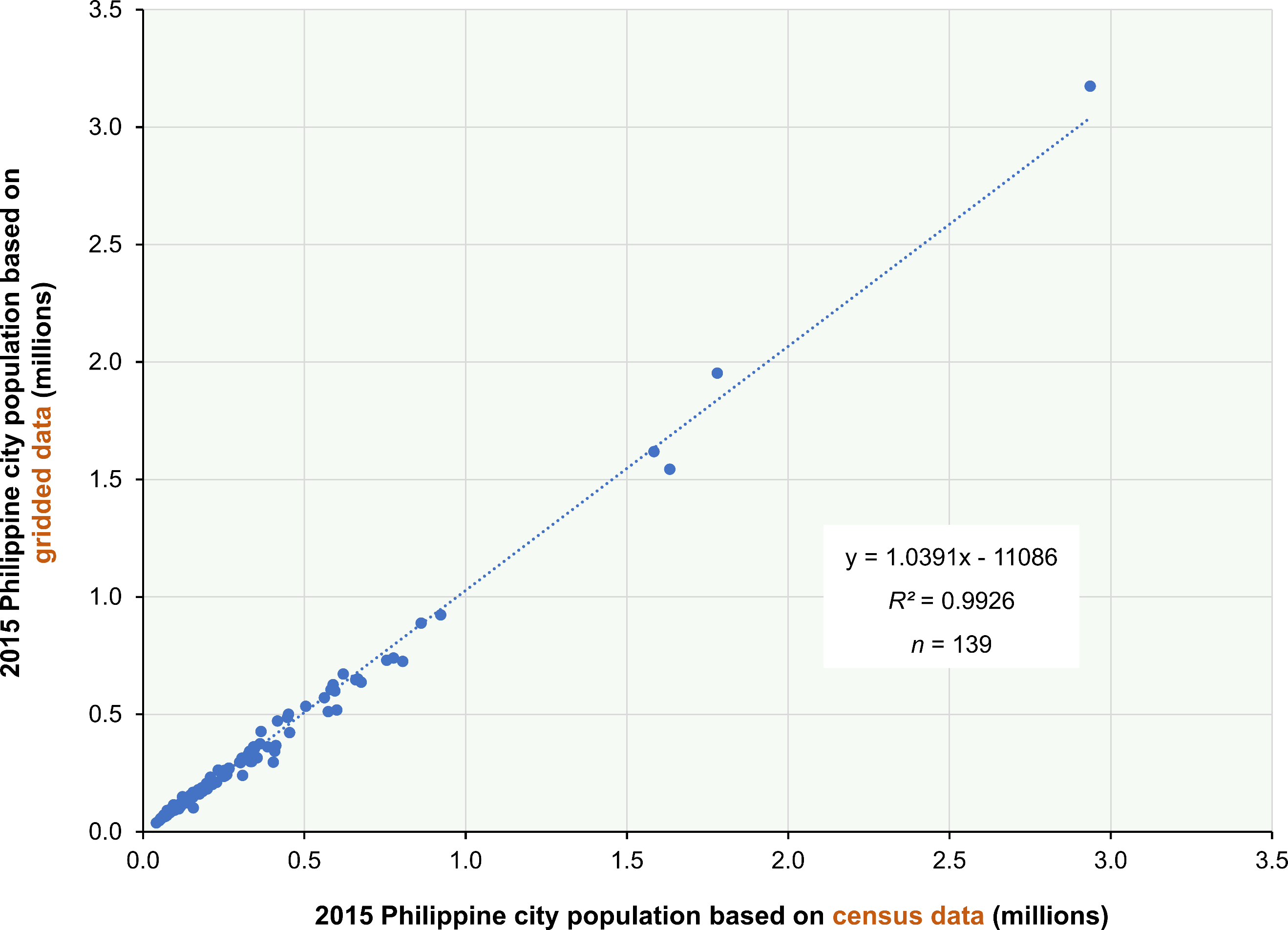


**Supplementary Figure 9** Scatter plot between total city population derived from the 2015 gridded population data (y-axis)^2,3^ and total city population based on the 2015 census data (x-axis)^1^. The result shows a very high (nearly 1:1) correlation, indicating that the gridded population data are accurate enough to be used for analysis. Each point represents a city.

| **Supplementary Table 1** Components of risk and vulnerability and their respective indicators used in this study. | | | |
| --- | --- | --- | --- |
| **Risk components** | **Factors^a^** | **Indicators (units)^b^** | **Data used and sources** |
| - Hazard | Heat | Land surface temperature (LST) (°C) | 2014–2016 MODIS land surface temperature (LST) (daily, March–May, 1 km)^4^ |
| - Exposure | Exposed human population | Population density (number of people per hectare) | 2015 gridded population data (100 m)^2,3^ |
| - Vulnerability | | | |
| **Vulnerability components** | **Factors^a^** | **Indicators (units)^b^** | **Data used and sources** |
| - Sensitivity^e^ | Age structure | Proportion of young population (age: < 15 years) (%) | 2015 census data^c^ |
|  |  | Proportion of old population (age: ≥ 65 years) (%) | 2015 census data^c^ |
|  | Socioeconomic status | Poverty incidence (%)^d^ | 2012 municipal and city-level poverty estimates^5^ |
| - Capacity^f^ | Natural resource | Availability of urban green space (with the use of a vegetation index) (unitless) | 2015 MODIS enhanced vegetation index (EVI) (16-day interval, March–May, 250 m)^6^ |
|  | Socioeconomic resource | City net income (PhP) | 2015 data on city net income^7^ |
|  |  | Per capita net income (PhP) | 2015 data on city net income^7^ and 2015 census data^1^ |
| ^a^ These factors were not included in the aggregation process. They are included here to provide context to the various indicators.  ^b^ The scale or level of measurement of the indicators was either interval or ratio. Hence, it was possible to use only one normalization technique (i.e., min-max approach) for all the indicators. In cases where other indicators fall under the nominal or ordinal scale of measurement, another type of normalization procedure is needed^8^.  ^c^ Data available from [www.citypopulation.de](http://www.citypopulation.de)  ^d^ Poverty incidence refers to the proportion of the number of families (individuals) with per capita annual income less than the per capita poverty threshold^9^.  ^e^ Sensitivity refers to the susceptibility of a system (e.g., a social-ecological system) or its elements (e.g., human) to harm and is determined by those factors that directly affect the consequences of a hazard, including physical, social and cultural attributes of a system or its elements^10,11^.  ^f^ Capacity includes coping capacity and adaptive capacity. Coping capacity refers to “the ability of people, institutions, organizations, and systems, using available skills, values, beliefs, resources, and opportunities, to address, manage, and overcome adverse conditions in the short to medium term” [p. 1762]^12^, whereas adaptive capacity refers to “the ability of systems, institutions, humans and other organisms to adjust to potential damage, to take advantage of opportunities, or to respond to consequences” [p. 1758]^12^. | | | |

| **Supplementary Table 2** Relative weights derived from the 12 expert (academics and researchers) questionnaire responses that had analytic hierarchy process (AHP) consistency ratios of <10%. | | | | | | | | | | | | | | |
| --- | --- | --- | --- | --- | --- | --- | --- | --- | --- | --- | --- | --- | --- | --- |
| **Risk components** | **AHP weights** | | | | | | | | | | | |  |  |
|  | **1** | **2** | **3** | **4** | **5** | **6** | **7** | **8** | **9** | **10** | **11** | **12** | **Average** | **SD** |
| Hazard | 0.10 | 0.65 | 0.16 | 0.08 | 0.07 | 0.06 | 0.10 | 0.13 | 0.33 | 0.13 | 0.09 | 0.74 | 0.22 | 0.23 |
| Exposure | 0.64 | 0.07 | 0.30 | 0.66 | 0.68 | 0.60 | 0.64 | 0.60 | 0.33 | 0.46 | 0.32 | 0.08 | 0.45 | 0.22 |
| Vulnerability | 0.26 | 0.28 | 0.54 | 0.26 | 0.25 | 0.35 | 0.26 | 0.28 | 0.33 | 0.42 | 0.59 | 0.18 | 0.33 | 0.12 |
| TOTAL | 1.00 | 1.00 | 1.00 | 1.00 | 1.00 | 1.00 | 1.00 | 1.00 | 1.00 | 1.00 | 1.00 | 1.00 | 1.00 | 0.00 |
| CR (%) | 4.0 | 6.8 | 1.0 | 3.4 | 1.0 | 2.3 | 4.0 | 0.6 | 0.0 | 1.0 | 1.0 | 4.6 | 2.48 | 2.07 |
| **Vulnerability components** | **AHP weights** | | | | | | | | | | | |  |  |
|  | **1** | **2** | **3** | **4** | **5** | **6** | **7** | **8** | **9** | **10** | **11** | **12** | **Average** | **SD** |
| Sensitivity | 0.20 | 0.88 | 0.33 | 0.86 | 0.13 | 0.14 | 0.17 | 0.83 | 0.50 | 0.25 | 0.80 | 0.17 | 0.44 | 0.32 |
| Capacity | 0.80 | 0.13 | 0.67 | 0.14 | 0.88 | 0.86 | 0.83 | 0.17 | 0.50 | 0.75 | 0.20 | 0.83 | 0.56 | 0.32 |
| TOTAL | 1.00 | 1.00 | 1.00 | 1.00 | 1.00 | 1.00 | 1.00 | 1.00 | 1.00 | 1.00 | 1.00 | 1.00 | 1.00 | 0.00 |
| **Sensitivity indicators** | **AHP weights** | | | | | | | | | | | |  |  |
|  | **1** | **2** | **3** | **4** | **5** | **6** | **7** | **8** | **9** | **10** | **11** | **12** | **Average** | **SD** |
| Young population | 0.08 | 0.07 | 0.12 | 0.19 | 0.07 | 0.07 | 0.48 | 0.14 | 0.23 | 0.13 | 0.07 | 0.73 | 0.20 | 0.20 |
| Old population | 0.19 | 0.65 | 0.61 | 0.08 | 0.68 | 0.68 | 0.45 | 0.33 | 0.08 | 0.19 | 0.28 | 0.19 | 0.37 | 0.24 |
| Poverty incidence | 0.73 | 0.28 | 0.27 | 0.73 | 0.25 | 0.25 | 0.07 | 0.53 | 0.70 | 0.69 | 0.65 | 0.08 | 0.44 | 0.26 |
| TOTAL | 1.00 | 1.00 | 1.00 | 1.00 | 1.00 | 1.00 | 1.00 | 1.00 | 1.00 | 1.00 | 1.00 | 1.00 | 1.00 | 0.00 |
| CR (%) | 6.8 | 6.8 | 7.7 | 6.8 | 1.0 | 1.0 | 0.3 | 5.6 | 8.0 | 9.8 | 6.8 | 6.8 | 5.62 | 3.10 |
| **Capacity indicators** | **AHP weights** | | | | | | | | | | | |  |  |
|  | **1** | **2** | **3** | **4** | **5** | **6** | **7** | **8** | **9** | **10** | **11** | **12** | **Average** | **SD** |
| Green space | 0.64 | 0.74 | 0.14 | 0.77 | 0.73 | 0.27 | 0.72 | 0.32 | 0.26 | 0.10 | 0.16 | 0.73 | 0.46 | 0.28 |
| City income | 0.10 | 0.17 | 0.53 | 0.07 | 0.20 | 0.06 | 0.09 | 0.09 | 0.10 | 0.47 | 0.54 | 0.20 | 0.22 | 0.18 |
| Per capita income | 0.26 | 0.09 | 0.33 | 0.16 | 0.07 | 0.67 | 0.19 | 0.59 | 0.64 | 0.43 | 0.30 | 0.07 | 0.32 | 0.22 |
| TOTAL | 1.00 | 1.00 | 1.00 | 1.00 | 1.00 | 1.00 | 1.00 | 1.00 | 1.00 | 1.00 | 1.00 | 1.00 | 1.00 | 0.00 |
| CR (%) | 4.0 | 1.5 | 5.6 | 5.6 | 1.0 | 3.0 | 9.8 | 1.0 | 4.0 | 0.6 | 1.0 | 9.8 | 3.91 | 3.28 |
| Note: Face-to-face expert consultation was conducted in March 2019. The last AHP pairwise comparison survey questionnaire was received on 21 June 2019. A total of 26 experts were asked to respond to the questionnaire. All except two responded. Of the 24 retrieved questionnaires, 12 had CRs of <10% for the risk components, sensitivity indicators, and capacity indicators. These were used in the analysis and are numbered in this table and Supplementary Table 3 as 1, 2, 3, and so on up to 12. Details about AHP can be found in various sources^13,14^ (see also https://people.revoledu.com/kardi/tutorial/AHP/, <https://bpmsg.com/academic/ahp.php>). In the questionnaire, a request letter was included to obtain consent from the experts to participate in the study. SD – standard deviation | | | | | | | | | | | | | | |

| **Supplementary Table 3** Details of the 12 experts whose questionnaire responses had analytic hierarchy process (AHP) consistency ratios (CRs) of <10%. | |
| --- | --- |
| **Expert number** | **Specialization/research themes** |
| 1 | Climate change vulnerability and adaptation; Disaster management; Social-ecological systems; Remote sensing; Urban and environmental geography |
| 2 | Urban climate change; Urban heat islands; Remote sensing; Urban and environmental geography |
| 3 | Medicine and public health; Environmental public health |
| 4 | Urban and environmental geography; Urban heat islands; Remote sensing; Geospatial analysis |
| 5 | Climate risk, hazard, and vulnerability |
| 6 | Forestry and environmental sciences |
| 7 | Urban and environmental geography; Urban heat islands; Remote sensing; Geospatial analysis |
| 8 | Capacity building; Geospatial solutions; Urban and environmental geography; Remote sensing; Geospatial analysis |
| 9 | Climate change adaptation, resilience, and mitigation; Climate-change-related policies and programs; Forestry and environmental sciences; Agroforestry |
| 10 | Urban and environmental geography; Urban heat islands; Remote sensing; Geospatial analysis |
| 11 | Geographical sciences and urban planning; Sustainability; Disaster assessment, recovery, and monitoring; Remote sensing; Geospatial analysis |
| 12 | Urban environmental geography; Urban heat island; Remote sensing; Geospatial analysis |
| Note: Face-to-face expert consultation was conducted in March 2019. The last AHP pairwise comparison survey questionnaire was received on 21 June 2019. A total of 26 experts were asked to respond to the questionnaire. All except two responded. Of the 24 retrieved questionnaires, 12 had CRs of <10% for the risk components, sensitivity indicators, and capacity indicators. These were used in the analysis and are numbered in this table and Supplementary Table 2 as 1, 2, 3, and so on up to 12. Details about AHP can be found in various sources^13,14^ (see also https://people.revoledu.com/kardi/tutorial/AHP/, <https://bpmsg.com/academic/ahp.php>). In the questionnaire, a request letter was included to obtain consent from the experts to participate in the study. | |

| **Supplementary Table 4** Derived heat health risk index (HHRI) values for Philippine cities. | | | | | | | | | | | | | |
| --- | --- | --- | --- | --- | --- | --- | --- | --- | --- | --- | --- | --- | --- |
| **City name** | **HHRI** | **Rank** | **95% CI** * |  | **City name** | **HHRI** | **Rank** | **95% CI** * |  | **City name** | **HHRI** | **Rank** | **95% CI** * |
| Manila City | 0.808 | 1 | 0.711–0.905 |  | Cabuyao City | 0.327 | 48 | 0.254–0.401 |  | Pagadian City | 0.234 | 95 | 0.164–0.305 |
| Mandaluyong City | 0.644 | 2 | 0.559–0.729 |  | El Salvador City | 0.323 | 49 | 0.238–0.408 |  | Kidapawan City | 0.233 | 96 | 0.144–0.322 |
| Caloocan City | 0.639 | 3 | 0.566–0.712 |  | Bais City | 0.321 | 50 | 0.237–0.405 |  | Malaybalay City | 0.232 | 97 | 0.154–0.310 |
| Pasay City | 0.628 | 4 | 0.525–0.731 |  | Santa Rosa City | 0.320 | 51 | 0.237–0.403 |  | Ormoc City | 0.229 | 98 | 0.144–0.313 |
| Malabon City | 0.610 | 5 | 0.538–0.682 |  | Tuguegarao City | 0.319 | 52 | 0.227–0.410 |  | Naga City, Camarines Sur | 0.228 | 99 | 0.157–0.300 |
| San Juan City | 0.608 | 6 | 0.513–0.702 |  | Cotabato City | 0.318 | 53 | 0.245–0.390 |  | Tabaco City | 0.227 | 100 | 0.140–0.314 |
| Marikina City | 0.589 | 7 | 0.498–0.679 |  | Tacurong City | 0.318 | 54 | 0.236–0.400 |  | Iriga City | 0.225 | 101 | 0.131–0.319 |
| Las Piñas City | 0.561 | 8 | 0.457–0.665 |  | Urdaneta City | 0.317 | 55 | 0.230–0.403 |  | Cagayan de Oro City | 0.222 | 102 | 0.158–0.286 |
| Taguig City | 0.534 | 9 | 0.459–0.610 |  | Batac City | 0.314 | 56 | 0.213–0.415 |  | Mati City | 0.222 | 103 | 0.132–0.312 |
| Parañaque City | 0.526 | 10 | 0.409–0.643 |  | La Carlota City | 0.313 | 57 | 0.225–0.401 |  | Legazpi City | 0.221 | 104 | 0.138–0.305 |
| Valenzuela City | 0.511 | 11 | 0.423–0.599 |  | Muñoz City | 0.313 | 58 | 0.215–0.411 |  | Ligao City | 0.220 | 105 | 0.126–0.313 |
| Pasig City | 0.508 | 12 | 0.411–0.604 |  | Canlaon City | 0.307 | 59 | 0.219–0.396 |  | Digos City | 0.217 | 106 | 0.146–0.288 |
| San Pedro City | 0.479 | 13 | 0.391–0.567 |  | Dumaguete City | 0.307 | 60 | 0.230–0.385 |  | Toledo City | 0.216 | 107 | 0.156–0.276 |
| Bacoor City | 0.459 | 14 | 0.355–0.564 |  | Tanjay City | 0.305 | 61 | 0.221–0.390 |  | Batangas City | 0.215 | 108 | 0.147–0.284 |
| Quezon City | 0.447 | 15 | 0.357–0.537 |  | Roxas City | 0.303 | 62 | 0.229–0.378 |  | Lipa City | 0.212 | 109 | 0.141–0.283 |
| Muntinlupa City | 0.442 | 16 | 0.353–0.530 |  | Trece Martires City | 0.301 | 63 | 0.217–0.385 |  | Naga City, Cebu | 0.212 | 110 | 0.154–0.269 |
| Mandaue City | 0.423 | 17 | 0.349–0.496 |  | Malolos City | 0.300 | 64 | 0.223–0.377 |  | Oroquieta City | 0.212 | 111 | 0.119–0.305 |
| Makati City | 0.418 | 18 | 0.293–0.544 |  | Santiago City | 0.299 | 65 | 0.220–0.377 |  | Danao City | 0.211 | 112 | 0.133–0.288 |
| Angeles City | 0.392 | 19 | 0.288–0.497 |  | Dapitan City | 0.297 | 66 | 0.192–0.402 |  | Gingoog City | 0.210 | 113 | 0.113–0.307 |
| Meycauayan City | 0.387 | 20 | 0.292–0.483 |  | Valencia City | 0.290 | 67 | 0.211–0.370 |  | San Pablo City | 0.209 | 114 | 0.130–0.287 |
| San Carlos City, Pangasinan | 0.387 | 21 | 0.291–0.483 |  | Passi City | 0.289 | 68 | 0.205–0.374 |  | Baybay City | 0.208 | 115 | 0.110–0.306 |
| Imus City | 0.385 | 22 | 0.280–0.490 |  | Silay City | 0.289 | 69 | 0.207–0.370 |  | Sorsogon City | 0.206 | 116 | 0.113–0.300 |
| Laoag City | 0.378 | 23 | 0.268–0.488 |  | Cabanatuan City | 0.287 | 70 | 0.210–0.364 |  | Baguio City | 0.205 | 117 | 0.136–0.273 |
| Biñan City | 0.376 | 24 | 0.290–0.462 |  | Victorias City | 0.287 | 71 | 0.209–0.364 |  | Calapan City | 0.203 | 118 | 0.128–0.279 |
| Iloilo City | 0.375 | 25 | 0.300–0.449 |  | Ozamis City | 0.284 | 72 | 0.207–0.362 |  | Olongapo City | 0.197 | 119 | 0.130–0.264 |
| Bago City | 0.368 | 26 | 0.281–0.455 |  | Sipalay City | 0.279 | 73 | 0.198–0.359 |  | Tagum City | 0.195 | 120 | 0.124–0.265 |
| Cauayan City | 0.368 | 27 | 0.271–0.465 |  | Koronadal City | 0.274 | 74 | 0.199–0.349 |  | Bayugan City | 0.194 | 121 | 0.098–0.289 |
| Tarlac City | 0.367 | 28 | 0.267–0.467 |  | Tanauan City | 0.272 | 75 | 0.195–0.350 |  | Zamboanga City | 0.191 | 122 | 0.132–0.251 |
| Dasmariñas City | 0.366 | 29 | 0.287–0.445 |  | San Fernando City, La Union | 0.271 | 76 | 0.188–0.353 |  | Borongan City | 0.190 | 123 | 0.099–0.281 |
| Bayawan City | 0.364 | 30 | 0.277–0.451 |  | Dipolog City | 0.270 | 77 | 0.174–0.367 |  | Bislig City | 0.186 | 124 | 0.094–0.277 |
| Dagupan City | 0.364 | 31 | 0.291–0.437 |  | Masbate City | 0.267 | 78 | 0.182–0.352 |  | Cabadbaran City | 0.186 | 125 | 0.106–0.265 |
| General Santos City | 0.364 | 32 | 0.277–0.452 |  | Cadiz City | 0.263 | 79 | 0.184–0.343 |  | Catbalogan City | 0.186 | 126 | 0.104–0.268 |
| San Fernando City, Pampanga | 0.363 | 33 | 0.266–0.460 |  | Lucena City | 0.262 | 80 | 0.190–0.333 |  | Butuan City | 0.181 | 127 | 0.108–0.255 |
| Vigan City | 0.363 | 34 | 0.264–0.462 |  | Samal City | 0.262 | 81 | 0.170–0.354 |  | Calbayog City | 0.181 | 128 | 0.088–0.274 |
| Candon City | 0.362 | 35 | 0.264–0.459 |  | San Carlos City, Negros Occidental | 0.262 | 82 | 0.190–0.334 |  | Cebu City | 0.181 | 129 | 0.129–0.233 |
| Kabankalan City | 0.362 | 36 | 0.278–0.445 |  | Talisay City Cebu | 0.262 | 83 | 0.200–0.324 |  | Antipolo City | 0.177 | 130 | 0.107–0.247 |
| Bogo City | 0.360 | 37 | 0.272–0.449 |  | Balanga City | 0.259 | 84 | 0.184–0.334 |  | Tabuk City | 0.177 | 131 | 0.115–0.239 |
| Bacolod City | 0.359 | 38 | 0.270–0.448 |  | San Jose del Monte City | 0.259 | 85 | 0.191–0.328 |  | Panabo City | 0.175 | 132 | 0.108–0.242 |
| Alaminos City | 0.358 | 39 | 0.261–0.456 |  | Carcar City | 0.258 | 86 | 0.186–0.330 |  | Tacloban City | 0.170 | 133 | 0.089–0.251 |
| Palayan City | 0.355 | 40 | 0.269–0.441 |  | Mabalacat City | 0.256 | 87 | 0.179–0.334 |  | Iligan City | 0.162 | 134 | 0.084–0.240 |
| Gapan City | 0.354 | 41 | 0.262–0.447 |  | Tagbilaran City | 0.252 | 88 | 0.188–0.315 |  | Tagaytay City | 0.154 | 135 | 0.084–0.224 |
| San Jose City | 0.354 | 42 | 0.261–0.447 |  | Surigao City | 0.251 | 89 | 0.170–0.332 |  | Davao City | 0.148 | 136 | 0.084–0.211 |
| Escalante City | 0.346 | 43 | 0.260–0.432 |  | Maasin City | 0.245 | 90 | 0.135–0.355 |  | Tandag City | 0.139 | 137 | 0.062–0.215 |
| Lapu-Lapu City | 0.345 | 44 | 0.274–0.415 |  | Ilagan City | 0.244 | 91 | 0.167–0.322 |  | Tayabas City | 0.129 | 138 | 0.056–0.203 |
| Tangub City | 0.344 | 45 | 0.250–0.439 |  | Calamba City | 0.240 | 92 | 0.174–0.307 |  | Puerto Princesa City | 0.098 | 139 | 0.045–0.150 |
| Sagay City | 0.333 | 46 | 0.250–0.415 |  | Lamitan City | 0.238 | 93 | 0.147–0.329 |  |  |  |  |  |
| Himamaylan City | 0.328 | 47 | 0.249–0.407 |  | Talisay City, Negros Occidental | 0.238 | 94 | 0.165–0.312 |  |  |  |  |  |
| * 95% CI was derived using the R programming statistical package called Rmisc^15^.  Note: The six cities not included in the analysis were Navotas City (Metro Manila), Cavite City (Luzon), General Trias City (Luzon), Guihulngan City (Visayas), Isabela City (Mindanao) and Marawi City (Mindanao). These cities either lacked temperature data (no data as per the remote sensing data used) or had incomplete socioeconomic data. | | | | | | | | | | | | | |

**Supplementary References**

1. PSA. *2018 Philippine Statistical Yearbook*. (Philippine Statistics Authority, Metro Manila, Philippines, 2018).

2. Tatem, A. J. WorldPop, open data for spatial demography. *Sci. Data* **4**, 170004 (2017).

3. Lloyd, C. T. High resolution global gridded data for use in population studies. *High Resolut. Glob. gridded data use Popul. Stud.* **4**, 170001 (2017).

4. Wan, Z., Hook, S. & Hulley, G. *MOD11A1 MODIS/Terra Land Surface Temperature/Emissivity Daily L3 Global 1km SIN Grid V006 [Data Set]*. (NASA EOSDIS Land Processes DAAC, 2015).

5. PSA. *2012 Municipal and City Level Poverty Estimates*. *Philippine Statistics Authority* (Philippine Statistics Authority, Metro Manila, Philippines, 2016).

6. Didan, K. *MOD13Q1 MODIS/Terra Vegetation Indices 16-Day L3 Global 250m SIN Grid V006 [Data Set]*. (NASA EOSDIS Land Processes DAAC, 2015).

7. COA. *2015 Annual Finacial Report for Local Government*. (Commission on Audit, Metro Manila, Philippines, 2016).

8. Fritzsche, K. *et al.* *The vulnerability sourcebook: Concept and guidelines for standardised vulnerability assessments*. (GIZ, Bonn, 2014).

9. NSCB. *Refinements in the Official Poverty Estimation Methodology*. (National Statistical Coordination Board, Metro Manila, Philippines, 2011).

10. IPCC. Summary for Policymakers. in *Climate Change 2014: Impacts, Adaptation, and Vulnerability. Part A: Global and Sectoral Aspects. Contribution of Working Group II to the Fifth Assessment Report of the Intergovernmental Panel on Climate Change* (eds. Field, C. B. et al.) (Cambridge University Press, Cambridge, United Kingdom and New York, NY, USA, 2014).

11. GIZ & EURAC. *Risk Supplement to the Vulnerability Sourcebook*. (GIZ, Bonn, 2017).

12. IPCC. *Climate Change 2014: Impacts, Adaptation, and Vulnerability. Part B: Assessment, Regional Aspects. Contribution of Working Group II to the Fifth Report of the Intergovernmental Panel on Climate Change*. (Cambridge University Press, Cambridge, United Kingdom and New York, NY, USA, 2014).

13. Saaty, T. L. Decision making with the analytic hierarchy process. *Int. J. Serv. Sci.* **1**, 83–98 (2008).

14. Estoque, R. C. Analytic Hierarchy Process in Geospatial Analysis. in *Progress in Geospatial Analysis* (ed. Murayama, Y.) 157–181 (Springer Japan, 2012).

15. Hope, R. M. Rmisc: Rmisc: Ryan Miscellaneous. R package version 1.5. (2013).
