# Supplementary material for: Heat health risk assessment in Philippine cities using remotely sensed data and social-ecological indicators
Source: Nat Commun. 2020 Mar 27;11:1581. doi: 10.1038/s41467-020-15218-8 (PMC7101384; doi:10.1038/s41467-020-15218-8)
Supplement: Supplementary file 3 — Reporting Summary [file 41467_2020_15218_MOESM3_ESM.pdf]

nature research

Corresponding author(s): DBPR NCOMMS-19-33548A

Last updated by author(s): Feb 20, 2020

Reporting Summary

Nature Research wishes to improve the reproducibility of the work that we publish. This form provides structure for consistency and transparency in reporting. For further information on Nature Research policies, see Authors & Referees and the Editorial Policy Checklist.

Statistics

For all statistical analyses, confirm that the following items are present in the figure legend, table legend, main text, or Methods section.

n/a	Confirmed	
☐	☒	The exact sample size (*n*) for each experimental group/condition, given as a discrete number and unit of measurement
☐	☒	A statement on whether measurements were taken from distinct samples or whether the same sample was measured repeatedly
☐	☒	The statistical test(s) used AND whether they are one- or two-sided<br>*Only common tests should be described solely by name; describe more complex techniques in the Methods section.*
☐	☒	A description of all covariates tested
☐	☒	A description of any assumptions or corrections, such as tests of normality and adjustment for multiple comparisons
☐	☒	A full description of the statistical parameters including central tendency (e.g. means) or other basic estimates (e.g. regression coefficient) AND variation (e.g. standard deviation) or associated estimates of uncertainty (e.g. confidence intervals)
☐	☒	For null hypothesis testing, the test statistic (e.g. *F*, *t*, *r*) with confidence intervals, effect sizes, degrees of freedom and *P* value noted<br>*Give P values as exact values whenever suitable.*
☒	☐	For Bayesian analysis, information on the choice of priors and Markov chain Monte Carlo settings
☒	☐	For hierarchical and complex designs, identification of the appropriate level for tests and full reporting of outcomes
☐	☒	Estimates of effect sizes (e.g. Cohen's *d*, Pearson's *r*), indicating how they were calculated

Our web collection on statistics for biologists contains articles on many of the points above.

Software and code

Policy information about availability of computer code

Data collection	Firefox Browser 71.0 (64-bit) was used to download Online datasets, e.g. MODIS LST, EVI and air temperature data.
Data analysis	Software used: ArcMap 10.5, R version 3.5.3 and Microsoft Office 365 ProPlus. Please see also 'Implementation' section of the manuscript.

For manuscripts utilizing custom algorithms or software that are central to the research but not yet described in published literature, software must be made available to editors/reviewers. We strongly encourage code deposition in a community repository (e.g. GitHub). See the Nature Research guidelines for submitting code & software for further information.

Data

Policy information about availability of data

All manuscripts must include a data availability statement. This statement should provide the following information, where applicable:

- Accession codes, unique identifiers, or web links for publicly available datasets
- A list of figures that have associated raw data
- A description of any restrictions on data availability

The sources of all the data used are acknowledged in the Methods section and Supplementary Information document. The complete list of the cities with their derived HHRI values, as well as the 12 sets of relative weights used, are given in the Supplementary Information document.

Field-specific reporting

Please select the one below that is the best fit for your research. If you are not sure, read the appropriate sections before making your selection.

☐ Life sciences ☒ Behavioural & social sciences ☐ Ecological, evolutionary & environmental sciences

For a reference copy of the document with all sections, see nature.com/documents/nr-reporting-summary-flat.pdf

Behavioural & social sciences study design

All studies must disclose on these points even when the disclosure is negative.

Study description	This study focused on heat health risk index development. It employed a mix-method approach in the analysis (statistical analysis, remote sensing, expert judgment). Please see 'Methods' for details.
Research sample	(1) The intention was to include all cities in the Philippines (100% sampling), but due to data limitation, only 139 cities were included in the analysis. (2) The same is true with the correlation between the HHRI and the NDAH, but also due to data limitation, only 65 cities were included in the analysis. (3) A purposive sampling was employed to identify 26 experts as respondents (with 24 responses; 12 were screened out). Other studies have consulted 6 to 30 experts, hence the number of experts consulted in this current study is within the range considered in other studies. Please see 'Methods' for details.
Sampling strategy	Please see above.
Data collection	(1) LST, EVI, air temperature, relative humidity, population data and other socioeconomic data - downloaded online using Firefox Browser 71.0 (64-bit). (2) Anonymized mortality data - obtained through request from the Philippine Statistics Agency. (3) Expert judgments - retrieved using AHP questionnaire. Please see 'Methods' for details.
Timing	For the retrieval of expert judgments - face-to-face consultation was done in March 2019, and the last questionnaire was received on June 21, 2019.
Data exclusions	(1) Exclusions of other cities - due to data limitation. (2) Screening of expert judgments - using AHP protocol (i.e. to be excluded if consistency ratio is equal or greater than 10%). Please see 'Methods' for details.
Non-participation	Out of 26 experts invited, two did not respond.
Randomization	The identification and selection of experts was purposive.

Reporting for specific materials, systems and methods

We require information from authors about some types of materials, experimental systems and methods used in many studies. Here, indicate whether each material, system or method listed is relevant to your study. If you are not sure if a list item applies to your research, read the appropriate section before selecting a response.

Materials & experimental systems

n/a	Involved in the study	
☒	☐	Antibodies
☒	☐	Eukaryotic cell lines
☒	☐	Palaeontology
☒	☐	Animals and other organisms
☐	☒	Human research participants
☒	☐	Clinical data

Methods

n/a	Involved in the study	
☒	☐	ChIP-seq
☒	☐	Flow cytometry
☒	☐	MRI-based neuroimaging

Human research participants

Policy information about studies involving human research participants

Population characteristics	See above.
Recruitment	Purposive sampling based on expertise. Nevertheless, the responses were screened based on an established AHP protocol for inclusion or exclusion. Please see above and 'Methods' for details.
Ethics oversight	National Institute for Environmental Studies, Japan.

Note that full information on the approval of the study protocol must also be provided in the manuscript.
